# Implementing Social Determinants of Health Screening in US Emergency Departments

**DOI:** 10.1001/jamanetworkopen.2025.0137

**Published:** 2025-03-06

**Authors:** Stephanie Loo, Melanie Molina, N. Jia Ahmad, Maeve Swanton, Olivia Chen, Krislyn M. Boggs, Carlos A. Camargo, Margaret Samuels-Kalow

**Affiliations:** 1 Department of Emergency Medicine, Massachusetts General Hospital, Boston; 2 Department of Emergency Medicine, University of California, San Francisco; 3 Harvard Medical School, Boston, Massachusetts; 4 Harvard T.H. Chan School of Public Health, Harvard University, Boston, Massachusetts

## Abstract

**Importance:**

Screening for adverse social determinants of health (SDOH) in the emergency department (ED) may help reduce health disparities in underserved populations.

**Objective:**

To understand barriers and facilitators to screening, documenting, and addressing adverse SDOH in a diverse sample of US EDs.

**Design, Setting, and Participants:**

This qualitative study used in-depth interviews with leaders of a purposive sample of EDs across urban, rural, academic, and community settings who self-reported screening for adverse SDOH on a prior National Emergency Department Inventory (NEDI) USA survey. EDs that completed the 2022 NEDI-USA survey and reported adverse SDOH screening were eligible for recruitment. Eligible participants were interviewed in April to September 2023. Inductive thematic analysis was conducted from September 2023 to January 2024 to identify themes and concepts.

**Main Outcomes and Measures:**

Themes and concepts related to ED practices for adverse SDOH screening and referral.

**Results:**

From 77 eligible EDs, 27 leaders agreed to be interviewed, (18 [66.7%] female; mean [range] age, 44 [30 to 63] years; mean [range] time in current role, 3.25 [<1 to 12] years). Participants worked in a variety of leadership roles (eg, chair or medical, nursing, or operations director). Findings centered around heterogeneity in ED adverse SDOH screening and documentation practices; skepticism of utility of ED adverse SDOH screening and referral; drivers of ED adverse SDOH screening, such as regulatory mandates for the expansion of adverse SDOH screening; resource, staffing, and time constraints in adverse SDOH screening and linkage to services processes; and recommendations and suggestions for improving the implementation of ED adverse SDOH screening, such as tailoring validated tools to the ED context and ED stakeholder engagement in designing the screening process. Other suggestions included having additional dedicated screening staff, particularly social workers, and strengthening relationships with existing non-ED SDOH initiatives and community resources dedicated to addressing adverse SDOH.

**Conclusions and Relevance:**

This qualitative study of US EDs describes an overview of practices and challenges surrounding adverse SDOH screening and identified novel solutions and areas where more research is needed for the successful implementation of adverse SDOH screening in the ED setting. At the policy level, regulatory mandates instituting adverse SDOH screening should include provisions for funding to support patient needs identified by screening. Additional research on development and implementation of ED adverse SDOH screening programs is needed.

## Key Points

**Question:**

What are the barriers and facilitators to screening, documenting, and addressing adverse social determinants of health (SDOH) according to a diverse sample of US emergency departments (EDs)?

**Findings:**

In this qualitative study including 27 leaders from diverse US EDs reporting adverse SDOH screening, EDs reported heterogeneity in adverse SDOH screening and documentation practices, skepticism of utility of ED adverse SDOH screening and referral, and challenges regarding resources, staffing and time to screen and address adverse SDOH needs.

**Meaning:**

These findings highlight the need for additional research on development and implementation of ED adverse SDOH screening programs, as well as funding provisions to support patient needs within regulatory mandates instituting adverse SDOH screening.

## Teaser

This qualitative study describes barriers and facilitators to screening, documenting, and addressing adverse social determinants of health in a diverse sample of US emergency departments.

## Introduction

Emergency departments (EDs) in the US often act as a safety net for patients experiencing adverse social determinants of health (SDOH). The World Health Organization defines SDOH as “the conditions in which people are born, grow, live, work, and age” which are “shaped by the distribution of money, power, and resources.”^1^ Patients with adverse SDOH, such as low income, housing insecurity, or difficulty paying for utilities or accessing transportation, use the ED at rates higher than the general population.^2,3,4,5,6,7,8^ Furthermore, the ED often serves as a key health care access point for patients without primary care.^9^ This renders the ED a critical place to identify adverse SDOH and connect patients to resources that may address their needs.

Prior studies have described operational outcomes for screening and referral for adverse SDOH in primary care and outpatient clinics, such as feasibility and patient engagement with resources, but the impact on long-term health outcomes and health care utilization remains unclear.^10,11,12^ Screening and referrals for adverse SDOH, such as access to housing, food, utilities, and transportation, are not yet widely implemented in ED settings—even when mandated by state law^13^—and lag behind screening for other health concerns, such as substance use and mental health.^14^

The Centers for Medicare & Medicaid Services, Joint Commission, and National Committee for Quality Assurance have begun to develop inpatient quality metrics for adverse SDOH screening and linkage to services that will be tied to reimbursement and accreditation by 2025.^15^ However, little is known about how these quality standards will be implemented in the ED or how EDs across the nation are screening, documenting, and addressing adverse SDOH for their patient populations.^16^ The goal of this study was to understand barriers and facilitators to screening, documenting, and addressing adverse SDOH in a diverse, representative sample of US EDs.

## Methods

This qualitative study was reviewed and approved by the Mass General Brigham institutional review board, and all participants provided informed consent. This study follows all Consolidated Criteria for Reporting Qualitative Research (COREQ) reporting guidelines.

### Participant Selection

We conducted in-depth qualitative interviews with administrative leaders of a purposive sample of US EDs selected with attention to variation across urban, rural, academic, and community status. The sample was drawn from the 2022 National Emergency Department Inventory (NEDI) USA survey, an annual survey and comprehensive database of all nonfederal, nonspecialty US EDs.^17^ A sample of 280 EDs was selected for geographic diversity and to balance across academic and nonacademic EDs. Of 232 responders, 77 reporting adverse SDOH screening policies were eligible for the qualitative interview. Specifically, participants for this study were sampled from responding EDs that reported policies to screen for adverse SDOH, including housing instability, difficulty paying for utilities or transportation, or food insecurity. Domains such as intimate partner violence (IPV), safety in the home, and substance use were not included.

### Measurements

Eligible EDs were contacted through phone and email recruitment for a 1-time interview, having previously participated in a NEDI-USA survey or informed of how their ED’s response to the survey made them eligible for this study. Interviews were completed with a single person per ED. Recruiting and interviewing took place between April and September 2023 via telephone in the interviewer and interviewee’s places of employment. We consented participants verbally, including obtaining consent to record interviews and publish quotes, and compensated them for their time with a $50 gift card. Participants provided demographic information, including sex, age, race, and ethnicity. Race was categorized as American Indian or Alaskan Native, Asian, Black or African American, Native Hawaiian or other Pacific Islander, White, and other. Ethnicity was categorized as Hispanic, Latino, or Spanish origin and not of Hispanic, Latino or Spanish origin. We collected race and ethnicity data to assist with contextualizing findings. Interviews were recorded and professionally transcribed and deidentified. All transcripts were quality checked against the original recordings for accuracy and deidentification verification.

The interview guide (eMethods 1 in Supplement 1) was informed by the aims of the study and relevant literature^18,19^ and then developed by the authors, who have expertise in qualitative methods and social emergency medicine. The interview guide was piloted among the research team (M.M., M.S., O.C., K.M.B., and M.S.-K.) and refined to ensure clarity and comprehension. All interviews were conducted by M.S. and O.C., who were trained and under the supervision of S.L. and M.S.-K., until data saturation was achieved, as agreed by the study team.

### Analysis

We used an inductive approach to identify emerging themes and concepts related to ED practices for adverse SDOH screening and referral.^20^ A coding tree (eMethods 2 in Supplement 1) was developed and refined by the study team by analyzing the data without preconceived categories or theories. Each transcript was individually and independently coded by 2 members of the study team (S.L. and M.M.), with discrepancies adjudicated by a third team member (M.S.-K.). Interviews were coded and analyzed using Dedoose (University of California, Los Angeles). Analysis was conducted from September 2023 to January 2024.

## Results

Of 77 eligible EDs, 27 operational leaders (response rate, 35.1%) from 16 states agreed to be interviewed. Of these, 18 (66.7%) identified as female. There were 1 American Indian or Alaskan Native participant (3.7%), 1 Asian participant (3.7%), 1 Native Hawaiian or other Pacific Islander (3.7%), and 24 White participants (88.9%); 2 participants (7.4%) identified Hispanic, Latino, or Spanish origin and 25 participants (92.6%) did not. The mean (range) age of participants was 44 (30-63) years (median, 41 years), with a mean (range) of 3 (<1 to 12) years (median, 4 years) of employment in their respective leadership roles. Roles included chair, medical director, and nursing/operations director (Table 1). There were 18 urban EDs (66.7%), and 15 EDs (55.6%) were in community hospitals (eTable in Supplement 1). Interviews were a mean (range) of 32 (21-45) minutes. Notably, several sites interviewed (6 sites [22.2%]) did not have adverse SDOH screening questions in place as defined by the research team. These EDs instead screened for other domains that the team did not define as core adverse SDOH, such as substance use, safety in the home, or IPV. Findings centered around heterogeneity in ED adverse SDOH screening and documentation practices; skepticism of utility of ED adverse SDOH screening and referral; drivers of ED adverse SDOH screening, such as regulatory mandates for the expansion of adverse SDOH screening in the medical setting; resource, staffing, and time constraints in adverse SDOH screening and linkage to services processes; and recommendations and suggestions for improving the implementation of ED adverse SDOH screening (Table 2).

**Table 1.** Participant Demographics

Characteristic	No. (%)
Age, mean (range), y	44 (30-63)
Gender	
Female	18 (66.7)
Male	9 (33.3)
Race	
American Indian or Alaskan Native	1 (3.7)
Asian	1 (3.7)
Black or African American	0
Native Hawaiian or other Pacific Islander	1 (3.7)
Other	0
White	24 (88.9)
Ethnicity	
Hispanic, Latino, or Spanish origin	2 (7.4)
Not of Hispanic, Latino, or Spanish origin	25 (92.6)
Role in emergency department	
Chair, vice chair, chief	7 (25.9)
Medical or nursing or operations director	13 (48.1)
Other administrative leadership	5 (18.5)
Other clinical leadership	2 (7.4)
Years in role, mean (range)	3.25 (<1-12)

**Table 2.** Themes and Representative Quotations

Theme	Representative quotation
**Heterogeneity in ED adverse SDOH screening and documentation practices**	
Screening at intake	“So when they first arrive here, the registration staff collects their demographics, so then they’ll identify if they’re housed or unhoused then. So from the beginning, they’re screened there. And then once the physician interviews to their medical screening exam, then it’s part of their exam where they ask about their vaccination status. And then from the nursing end, once they’re discharging them [the patients], they [the nurses] want to make sure they address all those needs.” (Site 24, urban academic)
	“I think the triage nurses will ask the questions that are put before them. So I think, at some point, just from a global standpoint, we recognize that we can only ask so many questions at initial intake. And building in social determinants of health kind of all along the process has been the way that we’ve kind of mitigated placing all the burden on the triage nurse.” (Site 14, urban academic)
**Skepticism of utility of ED adverse SDOH screening and referral**	
Support for the utility of social needs screening, especially for clinicians understanding patient context	“I think that we all agree that social needs are important for the health of the patient. I do think that we get very tied up in other documentation regarding assessments of their cardiac and other things that need to be documented for what they’re here for. So I do think that social needs and social determinants by the clinician are sometimes kind of pushed to the bottom until we can determine if they’ll need any assistance from their new medical diagnosis or whatever it may be.” (Site 8, urban academic)
Concern on patient reluctance to report needs but overall generally positive or ambivalent regarding patient response to social needs screening practices	“When they’re unwilling when they don’t want the help, we can’t force it upon them. Some of it is much [sic] pride. Some of them don’t want to be helped. Some of them are afraid that they’re going to lose their independence if they introduce resources into their home, and they see it as their homes are cluttered or really messy or we identified hoarding. So they’re worried. And they’ve heard horror stories before in the past how hoarding was identified and somebody was forced to move, but that was just probably years ago even before—probably unrelated to hospital. More relates as a social issue that wasn’t brought to the ER. So I think misconceptions, misperceptions by patients, having unwillingness to change and to want the help are our barriers.” (Site 15, rural community)
	“I think it’s probably very individual [on how patients feel about their social needs being documented]. I’m an incredibly lucky person, who I have not ever had risk of being unhoused at least. And I think that for a lot of patients, there is understanding—I think there’s a sense of relief when they’re asked about it. I don’t know if that sense of relief translates to them wanting it in their medical records. Having conversations with some patients, they feel like they are going to be judged the next time that they come in for medical care if it’s documented, that they either are food insecure or unhoused. And part of that, I think, comes across when they don’t want to answer the question in the first place or there is a sense of embarrassment or whatnot that’s associated with it. On the other hand, I think some patients, like I said, there’s a sense of relief, and they’re like, ‘Finally, someone’s going to maybe be able to actually provide me with some help.’ However, I think given, nationally, we don’t do a good job of providing ongoing help for patients. I think we can give them lists of immediate resources, but being in a shelter for a prolonged period of time is not a great way for them to get ongoing help with, say, substance abuse disorder or get them access to other education or get them access to job opportunities or… So I think that there is a cynicism for patients that have either been unhoused or have food insecurity for prolonged periods of time, so they are fairly indifferent to being asked or being documented because I don’t think they feel like it makes any kind of a difference.” (Site 21, urban academic)
Limitations of screenings for subsets of patients, such as patients who may already be a part of the shelter system or are reluctant to change lifestyles, noting that the onus of responding truthfully to such screenings was entirely on the patient	“To me, that’s probably one of the more frustrating things is trying to help the homeless population, as many of them really aren’t wanting assistance getting back on their feet. They’re comfortable in their current lifestyle. The resources that we have to get you into shelters generally puts restrictions on your lifestyle. You can’t drink, do drugs, come and go as you please at most shelters. And so that limits their desire to go to a shelter.” (Site 12, urban academic)
	“Some people, I think, actually lie about it for a variety of different reasons… Some people, for whatever reason, I think—not that they want to be homeless, but they decline shelters and stuff like that. And so some people, for whatever reason, just prefer that approach or whatever, and so they’ll decline resources or lie that they are homeless or insecure. And I don’t know if they—for some of the individuals, it might be stigma attached to it. For other people, I think it’s just where they want to be at that time for whatever reason. So despite offering these other resources, that’s just their preferred place that they want to be at that time.” (Site 10, urban community)
**Drivers of ED adverse SDOH screening**	
Policy-driven	“So departmentally, again, it’s a state requirement, so it’s not really an option. Personally, I think this is one of the many unfunded governmental mandates where politicians get involved in health care and don’t really have a full understanding of the details and ramifications of the laws that they pass. I think there are some pros and cons to it, but largely, it creates more work, and I’m not sure that it makes any significant difference.” (Site 12, urban academic)
Internally driven	“It wasn’t an emergency department–led initiative. It kind of came from a higher administration. I believe it’s the patient experience office who had been initiating the social needs screening. And as far as I know, they didn’t solicit input from the physicians in the ED either, because I feel like I would have been one of the people that they reached out to if they had done that, or I would have been tasked with giving them some information. So I do feel like it’s one of those things where you’re not asking the stakeholders on the ground, like, ‘What do you need just with patients?’ So I do think—and I do think there’s always some harm in asking patients to disclose these factors about their lives when we’re not prepared to follow up with substantive resources for them—I think there’s harm in that. So I think a lot of that nuance was lost in the implementation of this.” (Site 22, urban academic)
Driven by community needs	“So our quality department is working on initiatives to address social determinants of health. In the community needs assessment, there were several things identified, and our facility wants to identify those people that are suffering from lack of medical care, lack of medication access, and things like that. So this is something that they have just started working on as far as making it a front-end question, especially in the ER. A lot of these people, if they’ve got a lack of access to medical care, they don’t have insurance, they’re going to show up at the ER before they’re going to show up at primary care. So our ER is probably the best place to start that.” (Site 18, rural community)
**Resources, staffing, and time**	
For institutions that had connections to staffing or resources, there were limits regarding the availability of that person’s availability and time	“I think most hospitals have limited resources of social workers. For the most part, we have a dedicated ED social worker 24/7. But like every other position in health care recruitment in the last 2 to 3 years, it’s been challenging. So there are times that we have gaps, especially overnight and weekends. And so we’re reliant on 1 social worker who may be covering the entire hospital, not just the ED. I don’t think that’s unique to us.” (Site 12, urban academic)
	“The hour of the day [makes it hard to assist patients with social needs in the ED]. So anything after 6 o’clock in the evening, they’re [the patient] here until everything opens up and you can support them. That’s really the most difficult, because after a certain time, everything shuts down… [a]ny services outside: social services, shelters. You can’t get anything or anybody after a certain time because they’re closed for the day. So now you have to wait until the morning until these services open up in the morning.” (Site 1, urban community)
Participants noted that limitations of any or all 3 would greatly hinder the adverse SDOH implementation, screening, and linkage process	“[The] hardest thing is that the need outstrips the availability.” (Site 14, urban academic)
	“We are not as well-staffed as I would like us to be. I’d like to have 3 nurses every shift, but we only have 2 most of the time. That’s what’s required. But the amount of patients that they [the nurses] have to see at certain time frames throughout the day probably decreases them from asking more than required questions at times just because they’re trying to see as many patients as they’re able to and get all their [the patients’] needs met. So they may not have the amount of time to spend asking questions that aren’t required.” (Site 5, rural community)
	“I think we need additional resources to be able to take the time to address all of those other social determinants of health. And the fact of the matter is, is our social work team does an absolutely fantastic job. And even though we’re incredibly lucky—we have 24/7 coverage [from the social work team]—it’s still not enough. They need a little bit more time to manage some of these other patients so they can sit and take the time to make the phone calls and really sit down with the patient to figure out what is and isn’t going to work. Because each patient’s situation is so unique. And one person needs transportation, and another person doesn’t need transportation. They need a care coordinator. Someone else needs a health advocate. So I think being able to sit down and understand what that unique position is and figuring out how they can address some of those issues takes a lot of time and takes a lot of effort. And when you only have 2 social workers on to cover the entire ED that oftentimes has 300 patients, they are going to… they’re going to preferentially cover the emergencies as opposed to what they view as a chronic issue.” (Site 21, urban academic)
**5. Recommendations and suggestions for improving adverse SDOH ED implementation**	
Tailoring efforts could include reducing the number of social need domains asked in the ED and using tablet devices or patient portals to allow patients to respond to questions during waiting times at their ED visit	“What would be immensely helpful is if we had iPads or some electronic way for the patients to kind of work on that screening while they’re being seen as a patient, instead of having to go through because it’s—the one we have in our [EMR] system anyway—it’s about 7 or 8 topics, and within each topic, there are even more questions. So it’s extremely time-consuming and kind of personal to just start asking in the ER. So if there was a tablet or some easy way that we could screen, I feel like we could reach more people and get them services easier.” (Site 6, urban academic)
Improving workflow processes so that prior responses to screening questions, if asked in different areas of the health care system (ie, primary care), could be viewable, thereby reducing duplicative efforts in screening patients in the ED	“Our EMR isn’t the best for inputting information. So a lot of it’s going to be handwritten information and then input later to the computer. We use a very archaic EMR called [redacted]. So now the [private vendor EMR] and the [government-affiliated EMR] that are much more tech-friendly, this one is not. So I think if we had a better EMR, we could identify trends sometimes. Currently, if somebody comes to the ER, we have to screen them all over again, which is fine to screen them, but however, if it’d be nice to have something where we get an alert, ‘This patient was here 3 weeks ago and at the time didn’t have enough medication or enough money to afford medication.’ This way we could prompt the person to do the screening, ‘Oh, by the way, I notice you were here at one time. You didn’t have enough money for medications. How has that been? How is that working out?’” (Site 6, urban academic)
Having additional dedicated screening staff, particularly social workers, or strengthening relationships with existing non-ED SDOH initiatives and community resources dedicated to addressing adverse SDOH.	“Getting a core group of stakeholders, including some patient and community perspectives, when building the program. I feel like the community engagement is key, and identifying stakeholders early and getting champions who can model what you want to see for others and pull others into the program is probably the most important thing, especially in the ED environment.” (Site 22, urban academic)
	“And so it’s really important to have a strong social work presence in the emergency department. And I think we’re lucky, because one of the only reasons we have 24/7 [social work] coverage is because we’re a trauma center, so being a level II trauma center necessitated social work. So I think that there should be a push to have a larger social work presence in all emergency departments, right? We need that social worker regardless of whether we’re a trauma center or not.” (Site 10, urban community)

Abbreviations: 24/7, 24 hours a day, 7 days a week; ED, emergency department; EMR, electronic medical record; ER, emergency room; exam, examination; SDOH, social determinants of health.

### Heterogeneity in ED Adverse SDOH Screening and Documentation Practices

Screening practices varied by ED, but 3 commonalities emerged. Social need questions were often embedded within triage and/or registration processes. For example, one respondent stated, “So when they [the patient] first arrive here, the registration staff collects their demographics, so then they’ll identify if they’re housed or unhoused then.” Participants spoke about clinicians having the capability to ask social need questions ad hoc, if needed, and at any point during the ED visit, eg, “From a global standpoint, we recognize that we can only ask so many questions at initial intake, and building in social determinants of health along the process has been the way that we’ve kind of mitigated placing all the burden on the triage nurse.” Any identified needs were typically addressed through consultation with social workers. However, social workers were generally available only during business hours, despite EDs operating 24 hours a day, 7 days a week. This limited availability diminished ED clinicians’ ability to address social needs. In terms of documentation, questions were often standardized within the electronic medical record (EMR) system via check boxes or free-text notes. However, documentation of identified adverse SDOH needs was challenging due to time constraints, patient acuity, and/or EMR system limitations. One respondent explained, “Our EMR isn’t the best for inputting information, so a lot of it’s going to be handwritten information and then input later to the computer.”

### Skepticism of Utility of ED Adverse SDOH Screening and Referral

Participants expressed mixed support for the utility of social needs screening, especially for clinicians who understood the importance of the patient social context. One respondent said, “We all agree that social needs are important for the health of the patient; I do think that social needs and social determinants by the clinician are sometimes kind of pushed to the bottom until we can determine if they’ll need any assistance from their new medical diagnosis.” Participants expressed concern on patient reluctance to report needs but were overall generally positive or ambivalent regarding patient response to adverse SDOH screening practices, such as “When [patients are] unwilling, when they don’t want the help, we can’t force it upon them. Some of it is much pride. Some of them don’t want to be helped. Some of them are afraid that they’re going to lose their independence.” Participants further noted screening challenges for subsets of patients, such as patients experiencing homelessness who were already acquainted with shelters, with one respondent saying, “Some people, I think, actually lie about it for a variety of different reasons… not that they want to be homeless, but they decline shelters and stuff like that.” Participants reported concerns of potential adverse psychological impacts on patients who are screened for adverse SDOH without subsequent provision of resources. For example one respondent said, “I do think there’s always some harm in asking patients to disclose these factors about their lives when we’re not prepared to follow up with substantive resources for them.”

### Drivers of ED Adverse SDOH Screening

Institutional and state policies were primary drivers of ED screening. One respondent reported frustration with such a policy: “It’s a state requirement, so it’s not really an option. Personally, I think this is one of the many unfunded governmental mandates where politicians get involved in health care and don’t really have a full understanding of the details and ramifications of the laws that they pass.” Other drivers of ED adverse SDOH screening originated from other areas of the hospital, with one respondent explaining, “It wasn’t an emergency department–led initiative. It kind of came from a higher administration. I believe it’s the patient experience office who had been initiating the social needs screening.” For other EDs, adverse SDOH screening was part of the EMR system (but not required or often used), part of research initiatives, or driven by community needs. For example, one respondent said, “Our quality department is working on initiatives to address social determinants of health. In the community-needs assessment, there were several things identified: people that are suffering from lack of medical care, lack of medication access, and things like that.

### Resource, Staffing, and Time Constraints in Adverse SDOH Screening and Linkage to Services Processes

Resources, staffing, and time were unanimously reported as needed for the successful implementation of adverse SDOH screening in ED settings. Even institutions that had social work coverage 24 hours a day, 7 days a week experienced staffing gaps, such as “I think most hospitals have limited resources of social workers. For the most part, we have a dedicated ED social worker 24/7. But, like every other position in health care recruitment in the last 2 to 3 years, it’s been challenging. So there are times that we have gaps, especially overnight and weekends.” Participants noted that limitations of resources, staffing, and time would greatly hinder the adverse SDOH screening and linkage to services process, such as “We could significantly improve it [adverse SDOH screening] if we had a social worker that would be associated within the hospital who could assist us in the process, but we just don’t have the resources.” Another participant noted “[The] hardest thing is that the need outstrips the availability [of resources].” Other leaders highlighted that even with social work coverage available 24 hours a day, 7 days a week, there was not enough time for social workers to thoroughly review and address each patient’s individual needs, “Even though we’re incredibly lucky—we have 24/7 [social work] coverage—it’s still not enough. They need a little bit more time to manage some of these other patients so they can sit and take the time to make the phone calls and really sit down with the patient to figure out what is and isn’t going to work. Because each patient’s situation is so unique... figuring out how they can address some of those issues takes a lot of time and takes a lot of effort.”

### Recommendations and Suggestions for Improving Adverse SDOH ED Implementation

Participants had several suggestions for improving the implementation of adverse SDOH screening in the ED, such as involving ED stakeholders in the creation of screening forms and workflows or tailoring validated adverse SDOH screening tools (eg, Protocol for Responding to & Assessing Patients’ Assets, Risks & Experiences,^21^ Accountable Health Communities Health-Related Social Needs Screening Tool^22^) to ED settings. Some specific suggestions included reducing the number of social need domains asked in the ED and leveraging tablet devices or patient portals to screen during waiting times. One respondent suggested, “What would be immensely helpful is if we had [tablets] or some electronic way for the patients to kind of work on that screening while they’re being seen as a patient.” Other participants felt prior responses to screening questions asked either in the ED or a different clinical setting (eg, primary care) should be viewable by ED clinicians, thereby reducing duplicative ED screening efforts. One respondent said, “It’d be nice to have something where we get an alert, ‘This patient was here 3 weeks ago and at the time didn’t have enough medication or enough money to afford medication.’ This way, we could prompt the person to do the screening, ‘Oh, by the way, I notice you were here at one time. You didn’t have enough money for medications. How has that been? How is that working out?’” Other suggestions included having additional dedicated screening staff, particularly social workers, or expanding ED access to existing SDOH initiatives in their health system and strengthen relationships with community organizations dedicated to addressing adverse SDOH. Additional major challenges and potential solutions to improve adverse SDOH screening and interventions in ED settings are outlined in Table 3.

**Table 3.** Identified Issues and Proposed Solutions

Identified issues	Proposed solutions	Relevant quotes
EMR system limitations	Invest in rural, community EDs that lack supports for major EMR vendors Implement EMR updates that allow staff to review prior SDOH responses	“Keeping [the adverse SDOH screener as] simple as possible, but hopefully, having as many resources to offer. I think any of the patients that come in are in a time of, obviously, stress and not a desirable place, being no one shows up to the ED on a good day. So just being mindful of where people are in life and providing as many resources as possible, so hopefully one of them will work for the needs of the patient.” (Site 25, urban academic)
Implementation decisions	Include ED leadership and staff in implementation Link to community resources and public sectors to identify community needs	“[EDs] should try to get out into the community for outreach programs, such as… [police departments], public health, and school systems, [that] always seem to know what your social needs are in your community. That would be good resources to start with as far as identifying what needs to be addressed in a community.” (Site 26, rural community)
Social worker availability	Increase social worker and navigator coverage Promote telehealth navigation services for EDs unable to staff social workers	“Well, it would be great if we had a social worker. We do have a case manager, that she’s able to do some things as far as helping family with resources to get their elderly loved ones placed in nursing homes and stuff like that.” (Site 26, rural community)
		“I think that we could significantly improve it if we had a caseworker, a social worker that would—sorry, a social worker that would be associated within the hospital who could assist us in the process, but we just don’t have, like I mentioned before, the resources.” (Site 11, rural community)
Adverse SDOH documentation is time consuming	Standardize ED adverse SDOH questionnaires to consist of key domains pertinent to the ED setting (ie, housing, food, utilities, and transportation) Technological fixes, eg, automate responses, pull recent responses from other care areas (primary care, inpatient) Process changes, eg, ask questions in the waiting or nonacute care areas	“I would say, keep [the screening tool] short and sweet. Don’t ask the patient overly too many questions, but get to the point and ask them out front what they have.” (Site 4, rural community)
		“Having a charting [EMR] system with a standardized form is really a good thing to have. And if it’s customizable, you can customize it for the area, which is nice. Every community is different. Like I said, we don’t have a lot of diversity here. So our form standardized for our area is really nice to have.” (Site 18, rural community)
Adverse SDOH policies are unfunded mandates	Include funding provisions for implementation of adverse SDOH data collection and resource provision in the ED Increase awareness of adverse SDOH–associated billing and coding measures	“Quite honestly, we need state support at a level that is not there yet. It’s going to come down to money. We need more shelter beds. In some of our rural communities… if somebody lives out in the country, the availability of services in the rural parts of the state are pretty poor.” (Site 14, urban academic)
		“I would probably say [EDs starting their own adverse SDOH screening] need to try and find out if the community has any kind of grants and things like that available, because I think that kind of is what allowed us to start the program. So there are a lot of different grant programs available for that, for the hospitals to work with the community to try and get those grant programs brought into their hospital.” (Site 8, urban academic)

Abbreviations: ED, emergency department; EMR, electronic medical record; SDOH, social determinants of health.

## Discussion

In this national qualitative study of geographically diverse EDs, ED leaders from 16 US states acknowledged the significance of identifying and addressing adverse SDOH, reporting heterogenous practices for adverse SDOH screening, documentation, and interventions and skepticism about the potential impact of such screening processes on patients, particularly given the significant barriers identified. Often driven by institutional and state policies, these adverse SDOH screening practices were limited by scarce resources, staffing, and time. Suggestions for improvements included standardized screening tools, dedicated staffing, and streamlined resource linkage processes. Our findings using a diverse sample of EDs expand on a 2024 study by Mazurenko et al^23^ that focused on a public hospital system in the Midwest and noted the inconsistencies in the availability, collection, use of adverse SDOH data in the ED setting, as well as the challenges of limited resources to address adverse SDOH.

Our study highlights some of the ED-specific challenges related to adverse SDOH screening and interventions. These challenges should not deter ED-based SDOH practices altogether, given that the ED remains a safety net for populations with high rates of unmet social needs and novel strategies for these populations are needed, as demonstrated by recent evaluations of the outpatient-based Accountable Health Communities model, which found gaps in community resource linkages.^7,24,25^ Rather, this underscores the need for research investigating alternative methods for screening and interventions that make efficient use of limited resources and reduce the cognitive load on ED clinicians and staff. Future directions may focus on using data that are available in the EMR to identify patients with adverse SDOH or offering resources to all patients regardless of adverse SDOH screening responses, as opposed to requiring patient self-report. Some of this work is already in progress, such as the use of generative artificial intelligence models to identify SDOH in clinical notes^26,27,28,29,30^ and resource provision initiatives that do not require eligibility criteria.^31,32,33^

Our findings also identified drivers of adverse SDOH screening and intervention, particularly policies that require adverse SDOH data collection within the health system. However, as noted by our participants, these policies or mandates are often unfunded. This makes it difficult, if not impossible, to implement them, especially among already resource-limited hospitals. If violating these mandates incurs a penalty, this could potentially exacerbate disparities by worsening the situation for hospitals that primarily serve populations at higher risk or with higher rates of adverse SDOH. Potential solutions include documentation strategies that enable clinicians to receive credit for addressing, not just identifying, patients’ adverse SDOH needs and providing reimbursements for adverse SDOH–associated Z codes.^34,35^

### Limitations

This study has some limitations. One limitations is the potential for desirability bias and lack of generalizability. However, our respondents represented a mix of urban, rural, academic, and community EDs across 16 US states, building on more limited sample sizes of research conducted on this topic. Several sites (6 of 27 sites) did not have adverse SDOH screening questions in place as defined by the research team, instead screening for other domains that the team did not define as core adverse SDOH, such as substance use, safety in the home, or IPV. However, this is in part a limitation of how the study defined ED-specific adverse SDOH domains, given that some screens may include these domains within their SDOH modules.

## Conclusions

The findings of this qualitative study provide an overview of adverse SDOH screening and intervention practices in US EDs, identifying major challenges and potential solutions to improve adverse SDOH screening and interventions in ED settings. Many of these challenges and suggestions align with established literature on adverse SDOH screening and documentation in the primary care setting, including involving associated clinical staff in preimplementation efforts and streamlining EMR technology to simplify adverse SDOH screening workflows.^36,37^ Proposed solutions from this study include addressing EMR system limitations; including ED leadership and staff in adverse SDOH screening implementation efforts; increasing the availability of social workers, navigators, and case managers to address identified needs; and reducing documentation burdens for an already overburdened ED staff. There is great opportunity for technology to alleviate some of the burden of screening and intervention of ED staff by identifying needs (via large language models within the EMR), assisting with documentation (artificial intelligence scribes), and automating resource referrals or prompting linkage to resource services. At the broader policy level, regulatory mandates instituting adverse SDOH screening should include provisions for funding to support patient needs identified by screening.

## Notes

Supplement 1. **eMethods 1.** Interview Guide **eMethods 2.** Coding Tree **eTable.** Interview Site Designation

Supplement 2.
Data Sharing Statement
