# Supplementary material for: Implementing Social Determinants of Health Screening in US Emergency Departments
Source: JAMA Netw Open. 2025 Mar 6;8(3):e250137. doi: 10.1001/jamanetworkopen.2025.0137 (PMC11886722; doi:10.1001/jamanetworkopen.2025.0137)
Supplement: Supplement 1. — eMethods 1. Interview Guide eMethods 2. Coding Tree eTable. Interview Site Designation [file jamanetwopen-e250137-s001.pdf]

Supplemental Online Content

Loo S, Molina M, Ahmad NJ, et al. Implementing social determinants of health screening in US emergency departments. *JAMA Netw Open*. 2025;8(3):e250137.
doi:10.1001/jamanetworkopen.2025.0137

eMethods 1. Interview Guide

eMethods 2. Coding Tree

eTable. Interview Site Designation

This supplemental material has been provided by the authors to give readers additional information about their work.

© 2025 Loo S et al. *JAMA Network Open*.

1. **Interview Guide**

I'd like to ask you some specific questions related to screening for adverse social determinants of health, like housing insecurity or food insecurity —for convenience, we will just call those social determinants of health or social needs.

Screenings
High Priority Section

**Background**	**Firstly, can you please describe the social needs that your ED screens for?**<br>(FOR REFERENCE, DO NOT READ TO RESPONDENT: Housing, food insecurity, difficulty obtaining transportation, trouble paying utilities. Let them talk about any other need they mention [e.g., legal access, childcare, safety]. If they mention mental health, substance use, or violence, let them go through the interview and then ask if they have anything similar for one of the specified social needs.)<br>□ Are there any other social determinants of health that you screen for?<br><br>□When did the program start?<br>□ How?<br><br>□ Who in your hospital or ED implemented the program?<br><br>□ Can you take us from start to finish on this screening process?<br><br>□ Is this part of a set form/protocol with specific questions?<br>□ Or does the provider ask questions based on the patient's answers?<br>□ What staff members are involved?<br>□ What are their roles (positions)?<br>□ *(If they say "we" screen)* Who is "we"?	**Probes**<br>Who?<br>What?<br>When?<br>Where?<br>How?<br><br>➔ Return to the top for next social need if necessary
**About the respondent**	**Do you personally screen for social needs?** (Alternatively, in addition to *[others who screen]*, do you also personally screen for social needs? FOR REFERENCE, DO NOT READ TO RESPONDENT: Housing, food insecurity, difficulty obtaining transportation, trouble paying utilities)<br><br>**□ (IF YES) When do you do so? (probe for at triage/in the exam room/during the clinical encounter)**<br><br>▪ In what context? (example)<br>(FOR REFERENCE, DO NOT READ TO RESPONDENT: For all patients v. only the ones I'm worried about, specific chief complaints or patient presentations (e.g. patients who come in cold overnight)<br>▪ Do you screen for all patients? If not, what makes it more likely that you screen a patient?<br>▪ How do you decide? Are there certain criteria?<br><br>**□ (IF NO) I know you said earlier that _________ (from background question) screens for social needs, when do they do so? (probe for at triage/in the exam room/during the clinical encounter)**	

© 2025 Loo S et al. *JAMA Network Open*.

	▪ In what context do they screen? (example) (FOR REFERENCE, DO NOT READ TO RESPONDENT: For all patients v. only the ones I'm worried about, specific chief complaints or patient presentations (e.g. patients who come in cold overnight)<br>▪ Do they screen for all patients? If not, what makes it more likely that they screen a patient?<br>▪ How do they decide? Are there certain criteria?	
**Feasibility**	What systems are currently in place to support your ability to screen for social needs?<br>□ (FOR REFERENCE, DO NOT READ: screening pathways, question prompts, social work access)<br><br>**What else might support your ability to screen for social needs?** (FOR REFERENCE, DO NOT READ: Funding, additional programming, community resources)<br>□ Can you give me an example of how this would help, compared to what your doing now?<br><br>□ What are some barriers to screening? (FOR REFERENCE, DO NOT READ TO RESPONDENT: Funding, additional programming, community resources)<br>□ Can you walk me through how this issue hinders your ED?	

Is there anything else I should know about how social needs are identified in your ED?

I'm now going to switch gears and ask you a little bit about documentation.

Documentation

**Initial question**	**When patient's social needs identified during clinical care, are they routinely documented?**	
**YES routinely documented**	Is it a policy for your ED to document identified social needs?<br>□ If YES: can you tell me more about this policy?<br><br>**Can you walk us through how you document these social needs?** (use the below questions as probes only if you do not get an answer)<br>□ When does documentation occur? (FOR REFERENCE, DO NOT READ TO RESPONDENT: During registration, after a positive screen, at the end of the encounter)<br><br>□ Who documents the social needs?<br><br>□ Where are they documented? Patient chart in EHR, paper chart, external database<br><br>□ If they are documented in the EHR, are they documented using<br>standardized fields or free text notes?	

© 2025 Loo S et al. *JAMA Network Open*.

	**If social needs are routinely documented, how is this information used?**<br><br>□ When?<br>How often?<br>By whom?<br><br>□ Are documented social needs updated by your ED team?<br>□ If so, how often? By whom?<br><br>□ Does everyone have access to patient social risk information that is documented?<br>□ If not, who specifically has access?<br><br>□ How is documented social risk information used?<br>□ If any action/intervention is performed in response to an identified patient social risk, is this action/intervention also documented?<br>□ How so?	
**NO, not routinely documented**	Can you describe the characteristics of situations where social needs would be documented?<br>□ How do you decide? Are there certain criteria?<br><br>□ what are some of the barriers to documentation? (FOR REFERENCE, DO NOT READ TO RESPONDENT: Lack of time, lack of awareness of the ability to document social needs, lack of staff, patients are uncomfortable with this documentation)<br>□ How does this barrier affect your ability to document?	
**Incentives**	□ Does it affect billing in any way?<br>(Examples: MA ACO requiring 80% of patients to be screened for hospital to be paid, new billing criteria allows for diagnosis or treatment limited by SDoH to be used to meet moderate complexity criteria, https://cdn.ymaws.com/californiaacep.org/resource/resmgr/files/other/vici a-8_elements_of_med.pdf)<br><br>□ At your hospital, are you aware of any financial incentives for documenting patient social needs? (If they already reported some incentives via billing, you could say, "In addition to the financial incentives you already shared related to billing, are you aware of other financial incentives for documenting patient social needs?")<br>□ How are these costs covered? (FOR REFERENCE, DO NOT READ: By the hospital, the system, outside source)	
**Opinions**	In the ED context, how do you feel about documenting patient social needs?<br>□ What do you feel are some of the benefits of documenting patient social needs?<br>□ Drawbacks of documentation?	

© 2025 Loo S et al. *JAMA Network Open*.

	□ As a provider, how do you feel about documenting patient social needs?<br>□ How do you think other providers feel about documenting patient social needs?<br>□ Do patients know that their social needs are being documented?<br>□ If yes: How do you think the patient feels about documenting their social needs?<br>□ If no: How do you think the patient would feel about documenting their social needs?<br><br>□ How do you think your community feels about your ED documenting patient social needs?	

Now I'm going to ask you a few questions about how your hospital or health system addresses these social needs.

<u>Addressing</u>

<u>High Priority Section</u>

**Initial question**	**Once a patient's social needs are identified, what is the next step?**<br>□ By whom?<br>□ What do you do?<br>□ Where do you do it? To where do you refer them?<br>□ How?<br>□ When?<br><br>Is this standard procedure for patients who screen positive?<br>□ If YES**:** How did your ED decide on these standardized responses?<br>□ If NO: Can you give me an example of how you may respond differently based on the patient?
**Resources**	**Have you ever given patients information about additional resources from the ED?**<br>□ Where did you find these resources?<br><br>**What are some things that make it hard to assist patients with social needs in the ED?**<br>□ What makes it hard to find or provide those resources?<br><br>**What are some things that would make it easier to assist patients with social needs in the ED?**<br>□ What might help you to find or provide these resources?<br><br>**What are some things that would make it easier to connect people in the ED with community resources?**<br>□ For social needs?
**Additional**	**What kind of information about patient social needs might be helpful to ED clinicians as they are making a care plan?**<br>(Example, if needed) If a patient with a lower extremity fracture is about to be discharged home – The EHR can prompt them to look for a shelter rather than discharge to the street if they are experiencing homeless<br>□ Can you give me an example of how that might change or influence a decision you make?

© 2025 Loo S et al. *JAMA Network Open*.

	**Would it be helpful to have those detailed in the EMR? In what way?**<br><br>**Who might use it/what might be hard/easy etc.**<br><br>**Do you think ED clinicians would find it helpful if your EHR included tools, such as a clinical decision support tool, that informed them of the patients' social needs as they are making the care plan?**<br><br>□ Why or why not?<br>□ What do you think your ED clinicians would think about this?

Moving on from just the ED, I'd like to know more about what is happening in your hospital and health system:

Context

**Hospital Wide**	**Are there hospital programs that are in place, outside of the ED, to screen for social needs?**<br>□ To document them?<br>□ To address them?
**System Wide**	**Is your ED part of a health system?**<br><br>**(if yes) Are there programs that are in the health system, to screen for social needs?**<br>□ To document them?<br>□ To address them?

Comments/Recommendations

**Additional recs**	Thank you so much. Finally, **are there** any other strategies for improving ED social screenings that you would like to tell us about?<br><br>What would you recommend to other EDs who are trying to start up their own screening programs for social needs?

© 2025 Loo S et al. *JAMA Network Open*.

2. Coding Tree

A. Drivers

A1. Regulatory + billing

A2. Systems pressures + technology

A3. Community role and needs

A4. Other (e.g. research)

B. Practices (Screening Processes)

B1. Who does SN screening

B2. Who receives SN screening

B3. What happens from SN screening results

B4. How do they record (Documentation)

B5. Other

C. Experience/expectation of response / Anticipated outcomes

C1. Patient perspectives

C2. Anticipated changes

C3. Compliance

C4. Cynicism

C5. Implications for change

D. Change over time

D1. Policy

D2. Billing

D3. Financial

D4. Crowding

D5. Absence of institutional memory

E. Challenges

E1. Design / systems / tech

E2. Resources / time / staffing / screening burden

E3. Knowledge

E4. Other

© 2025 Loo S et al. *JAMA Network Open*.

F. Advice for improvement / recommendations/ facilitators

G. Good quotes

© 2025 Loo S et al. *JAMA Network Open*.

eTable 1. Interview Site Designation

ID	Urban/Rural Status	Academic/Community Hospital
1	urban	community
2	urban	academic
3	urban	academic
4	rural	community
5	rural	community
6	urban	academic
7	urban	academic
8	urban	academic
9	urban	community
10	urban	community
11	rural	community
12	urban	academic
13	rural	community
14	urban	academic
15	rural	community
16	urban	community
17	rural	community
18	rural	community
19	rural	community
20	urban	academic
21	urban	academic
22	urban	academic
23	urban	community
24	urban	academic
25	urban	academic
26	rural	community
27	urban	community

© 2025 Loo S et al. *JAMA Network Open*.
