# Supplementary material for: Implementing Social Determinants of Health Screening in US Emergency Departments
Source: JAMA Netw Open. 2025 Mar 6;8(3):e250137. doi: 10.1001/jamanetworkopen.2025.0137 (PMC11886722; doi:10.1001/jamanetworkopen.2025.0137)
Supplement: Supplement 2. — Data Sharing Statement [file jamanetwopen-e250137-s002.pdf]

Data Sharing Statement

Loo. Implementing Social Determinants of Health Screening in US Emergency Departments. *JAMA Netw Open*. Published March 06, 2025. doi:10.1001/jamanetworkopen.2025.0137

Data
Data available: No

Additional Information
Explanation for why data not available: Given confidentiality concerns around qualitative interview data, we are unable to share transcripts but may share summary de-identified data pending reasonable request to study PIs.
